# Implementation of continuous temperature monitoring during perioperative care: a feasibility study

**DOI:** 10.1186/s13037-022-00341-w

**Published:** 2022-09-24

**Authors:** Judy Munday, David Sturgess, Sabrina Oishi, Jess Bendeich, Allison Kearney, Clint Douglas

**Affiliations:** 1 Centre for Healthcare Transformation & School of Nursing, Queensland University of Technology (QUT) and Royal Brisbane and Women’s Hospital, Brisbane, Queensland Australia; 2 The University of Queensland (UQ) and Surgical Treatment and Rehabilitation Service (STARS), Brisbane, Queensland Australia; 3 Department of Anaesthetics, Princess Alexandra Hospital, Brisbane, Queensland Australia; 4 Princess Alexandra Hospital, Brisbane, Queensland Australia; 5 Department of Anaesthetics, Princess Alexandra Hospital, Brisbane, Queensland Australia; 6 Centre for Healthcare Transformation & School of Nursing, Queensland University of Technology (QUT) and Metro North Hospital and Health Service, Brisbane, Queensland Australia

**Keywords:** Feasibility, Temperature, Thermoregulation, Continuous monitoring

## Abstract

**Background:**

Continuous body temperature monitoring during perioperative care is enabled by using a non-invasive “zero-heat-flux” (ZHF) device. However, rigorous evaluation of whether continuous monitoring capability improves process of care and patient outcomes is lacking. This study assessed the feasibility of a large-scale trial on the impact of continuous ZHF monitoring on perioperative temperature management practices and hypothermia prevention.

**Methods:**

A feasibility study was conducted at a tertiary hospital. Participants included patients undergoing elective surgery under neuraxial or general anesthesia, and perioperative nurses and anesthetists caring for patient participants. Eighty-two patients pre and post introduction of the ZHF device were enrolled. Feasibility outcomes included recruitment and retention, protocol adherence, missing data or device failure, and staff evaluation of intervention feasibility and acceptability. Process of care outcomes included temperature monitoring practices, warming interventions and perioperative hypothermia.

**Results:**

There were no adverse events related to the device and feasibility of recruitment was high (60%). Treatment adherence varied across the perioperative pathway (43 to 93%) and missing data due to electronic transfer issues were identified. Provision of ZHF monitoring had most impact on monitoring practices in the Post Anesthetic Care Unit; the impact on intraoperative monitoring practices was minimal.

**Conclusions:**

Enhancements to the design of the ZHF device, particularly for improved data retention and transfer, would be beneficial prior to a large-scale evaluation of whether continuous temperature monitoring will improve patient outcomes. Implementation research designs are needed for future work to improve the complex area of temperature monitoring during surgery.

**Trial registration:**

Prospective registration prior to patient enrolment was obtained from the Australian and New Zealand Clinical Trials Registry (ANZCTR) on 16^th^ April 2021 (Registration number: ACTRN12621000438853).

## Background

Core body temperature alterations during surgery increase the risk of morbidity and mortality [1–3]. Frequent perioperative temperature monitoring enables proactive recognition of patients at-risk or already hypo/hyperthermic [3–5]. Yet pre and intraoperative temperature is rarely monitored in accordance with guidelines [3–6]. There is a high incidence of perioperative hypothermia in Australian facilities [6, 7], but only 20-31% of patients have intraoperative temperature monitoring [6–8]. The risk of death from malignant hyperthermia is also closely related to temperature monitoring practices, reaching 30% of cases when temperature goes unmonitored [1, 2].

In contrast to other vital signs, there has been a lack of non-invasive continuous temperature monitoring devices with clinically acceptable accuracy [9, 10] and intraoperative accessibility of data [11]. Various devices with different degrees of measurement accuracy are typically used with a single patient perioperatively. Recently, non-invasive devices providing continuous temperature monitoring across the perioperative pathway have become widely available, including the 3M™ Bair Hugger™ Temperature Monitoring System (3M™, St Paul, MN) “zero-heat-flux” (ZHF) device [12–15]. In 2020, a meta-analysis indicated that the ZHF device may be insufficiently accurate for use where wide temperature variations are anticipated [16]. Nonetheless, the device is no less accurate than non-invasive devices commonly used, such as aural canal devices. The ZHF device is easily attached and may be used for both awake and anesthetized patients. Patient acceptability is high [17]. As a continuous non-invasive device, it may increase consistency of perioperative temperature monitoring.

Rigorous evaluation is needed to determine whether continuous monitoring with easy, visible access to temperature data results in improved process of care and patient outcomes [15]. Intraoperative decision-support tools and electronic alerts have been found to support improvements in perioperative clinical care and documentation, including perioperative hypothermia prevention [11]. Lakha and colleagues identified that intraoperative decision-support and electronic alerts improved perioperative hypothermia prevention, but specifically related to compliance with quality measures (Measure #424, Perioperative Temperature Management) when linked to payment bonuses for anesthesiologists [11]. In the absence of payment incentives, whether continuous capability and easily accessible temperature data translates to improved practices and patient outcomes is unclear [15].

The first step in testing and implementation of continuous perioperative temperature monitoring with ZHF devices is to establish their acceptability and feasibility. Feasibility studies are used in preparation for large-scale trials to assess recruitment, protocol fidelity and process evaluations [18]. This study assessed the feasibility of a future trial on the impact of continuous monitoring on temperature management practices and hypothermia prevention.

## Methods

### Study design

This study assessed feasibility of recruitment and sampling methods, protocol fidelity, and intervention feasibility and acceptability. The study was conducted in a tertiary referral, urban public hospital in Australia. Data were collected from 2nd June 2021 to 1st April 2022. Secondary process of care outcomes included temperature monitoring practices as documented and as per guidelines, use of active warming strategies, and prevention of perioperative hypothermia. Ethical, site-specific, and administrative ethical approvals were obtained from the hospital and university’s Institutional Review Boards respectively (HREC/2021/QMS/70450; QUT 2021-3904-4302). Written informed consent was obtained from all participants. Prospective registration prior to patient enrolment was obtained from the Australian and New Zealand Clinical Trials Registry (ANZCTR) on 16^th^ April 2021 (Registration number: ACTRN12621000438853, Principal Investigator: J Munday). The study is reported according to the CONSORT extension for pilot and feasibility trials [19].

### Inclusion and exclusion criteria

Eligible patients included consenting adults undergoing elective surgery (of expected duration >30 minutes), with general or neuraxial anesthesia, with an American Society of Anesthesiologists (ASA) physical status score I-III. Patients were excluded if they had a known sensitivity to adhesives, forehead rash on the day of admission, or if admission to the Intensive Care Unit (ICU) directly from the perioperative department was planned. Eligible health care professionals included consenting perioperative registered nurses (RNs) and anesthesiologists caring for patient participants.

After written informed consent, data were prospectively collected from 42 patients before and 40 after the introduction of the ZHF device (82 patients in total). In the interim period between the before and after data collection periods, 20 patients received continuous temperature monitoring via the ZHF while the device was introduced into the department and training in the use of the device was undertaken. These patients are not included in the analysis. This sample size was sufficient to evaluate the feasibility of study methods [20].

### Study procedures

#### Usual care group

The first 42 patients enrolled in the trial received usual care. Temperature monitoring at all timepoints, from admission into the Surgical Care Unit (SCU) – referred to as the preoperative phase – to transfer to the anesthetic room, on induction, during surgery and during Post Anesthetic Care Unit (PACU) admission, was unchanged. Health care professionals were able to select whether to apply monitoring or not, and no direction as to the method of monitoring was provided.

The usual mode of preoperative temperature monitoring is via a Welch and Allyn™ oral device. This monitor can automatically transfer data to the electronic medical record (EMR), before patient transfer to the anesthetic room. Intraoperatively, the Philips™ anesthetic monitor can continuously monitor temperature if a device is attached and automatically transfers data into the EMR. In PACU, a Covidien™ tympanic temperature device is used and data manually entered into the EMR. However, the Philips™ monitor used in PACU can continuously monitor and record temperature at specific intervals with automatic data transfer into the EMR.

#### During the interim training period

The 3M™ Bair Hugger™ Temperature Monitoring System (Model 37000; 3M™, St Paul, MN) ZHF device was introduced into the department, and perioperative staff received training on the use of the device. For the 20 patients enrolled in the study during this period, clinicians received support in the use of the device by a research team member.

#### During the intervention period (ZHF Group)

The study protocol required that, on arrival to the Surgical Care Unit, and during the preoperative check-in process, the ZHF device was attached to the forehead (3M™ Bair Hugger™ Temperature Monitoring Patient Sensor, Model 36000), above the orbital ridge by the RN working in the Surgical Care Unit. After placement, the device requires a ramp-up time of up to three minutes for temperature readings to stabilize. The protocol specified that the device was to remain in-situ and available for use throughout transfer to the anesthetic room, on induction and during surgery, until patients were assessed as ready for discharge from PACU. Intraoperatively and in PACU, clinicians were able to plug the ZHF device into the anesthetic or PACU monitor, and data were transferred into the EMR in use at the study hospital. The device itself also displays the current temperature reading, and retrospective temperature data (up to two hours) is provided only as a trend (with no raw values).

### Primary and secondary outcome measures

Feasibility outcomes included the numbers of patients screened and recruited (assessed via recruitment log), aiming to achieve a consent rate of >50%, retention and treatment adherence (>80%) and missing data (<10%). Device failure and other adverse events were also recorded.

Intervention feasibility and acceptability [21] was measured by staff survey distributed via email, containing a link to the REDCap™ survey at the conclusion of the patient data collection period. The survey used the Acceptability of Intervention Measure (AIM); Intervention Appropriateness Measure (IAM) and Feasibility of Intervention Measure (FIM) tool developed by Weiner [22]. The tool measures the constructs (acceptability appropriateness and feasibility) on a five-point scale (*completely disagree, disagree, neutral – neither agree or disagree, agree, completely agree),* with higher scores indicating greater agreement with each corresponding statement related to acceptability, appropriateness and feasibility. The research team, in a prior study, assessed acceptability [21] from the perspective of patients, and side effects [17]. Practicality was assessed via a five-point Likert scale (*poor, fair, good, very good, excellent*) as used in prior studies assessing practicality of interventions [23].

Process of care outcomes included documented temperature measurement at key time points, active warming (including warmed intravenous fluids, forced air warming or other interventions), and appropriateness of warming (assessed against temperature and as per guidelines for prevention of perioperative hypothermia) [3, 4, 24]. Perioperative hypothermia was defined as a temperature <36.0°C [3] and was assessed if temperature data was documented in the medical record. The key time points used for temperature, hypothermia and appropriateness of warming assessments matched evidence-based guidelines for perioperative hypothermia prevention [3, 4].

Basic demographic data for patients included age, gender, surgical specialty, ASA score, body mass index (BMI), duration of time in preoperative holding area, surgical duration, PACU duration from admission until ‘ready for discharge’ assessment. Staff respondents indicated their professional role.

Data collection for feasibility and process of care outcomes was performed by an experienced, non-blinded research team member directly from the EMR, however treatment adherence was assessed prospectively, in real-time.

### Data analysis

Sample characteristics and feasibility outcomes (including acceptability, appropriateness, and practicality) are summarized as counts and percentages, medians and IQRs, as appropriate. Mann-Whitney and Fisher’s exact tests were used to compare pre and post group characteristics and change in process of care outcomes. Mean temperatures were computed by group over time and PACU admission temperature adjusted for baseline temperature and BMI, was compared before and after introduction of ZHF monitoring.

## Results

**Fig. 1:**
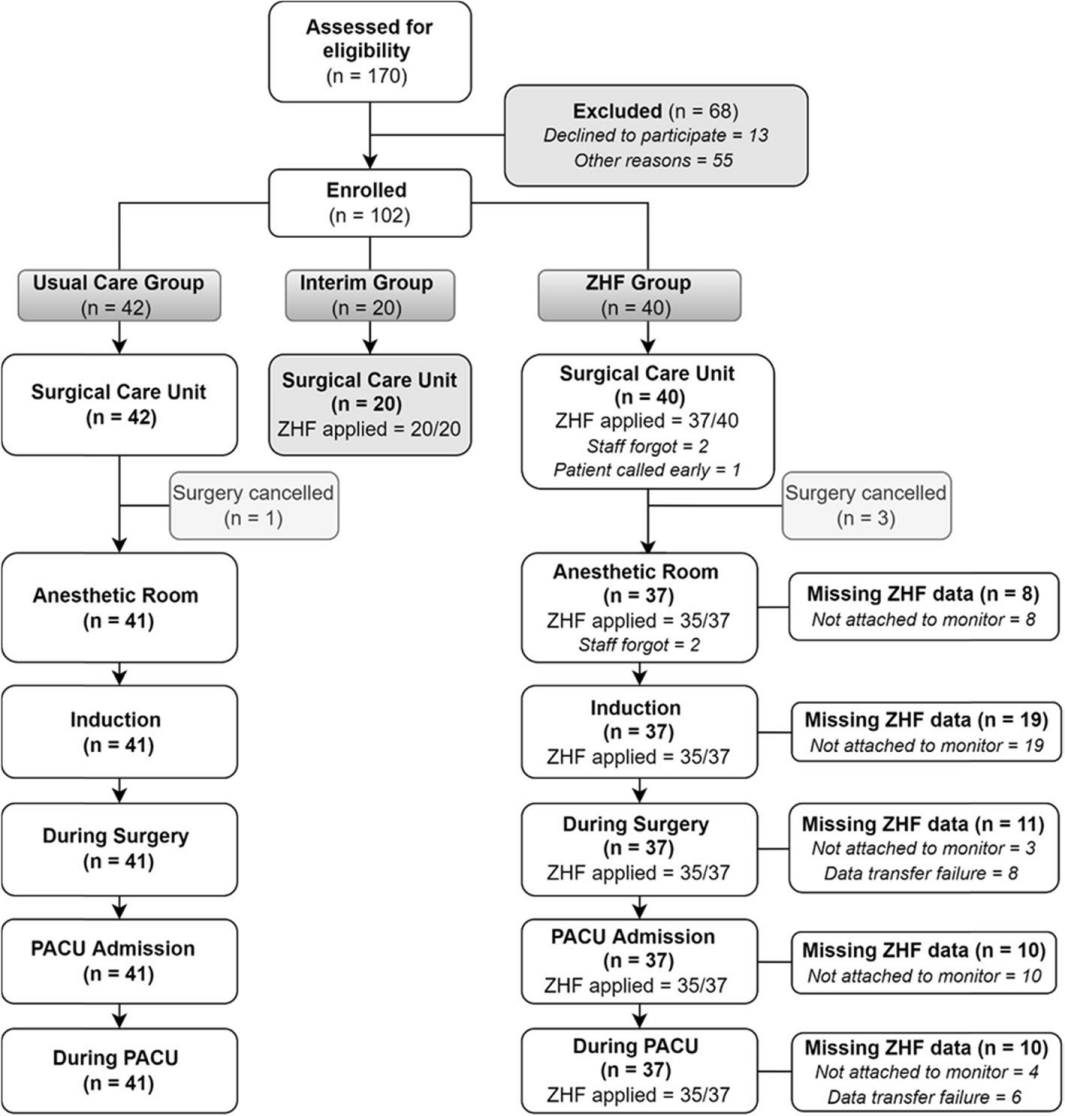
Participant flow, non-adherence, and missing data at each phase.* Abbreviations:* ZHF: zero-heat-flux

Of 102 patients enrolled, 42 were enrolled in the usual care group, 20 were enrolled in the interim (training period) group and 40 were enrolled in the ZHF group (see Fig. 1). All patient characteristics are reported in Table 1. Compared to the usual care group, the ZHF group had a lower BMI (*z* = 1.96, *p* = 0.05) which was likely to be clinically meaningful, and shorter PACU length of stay (*z* = 2.13, *p* = 0.03).

**Table 1:** Patient characteristics

**Characteristics**	**Usual care (*n* = 41)**	**ZHF (*n* = 37)**
**Age (years)**	59.2 (43.4-68.7)	62.0 (47.0-69.4)
**Body mass index**	30.9 (25.7-35.5)	27.7 (24.0-31.3)
**Gender**		
*Female*	18 (43.9)	13 (35.1)
*Male*	22 (53.7)	24 (64.9)
*Other/undisclosed*	1 (2.4)	0 (0)
**Type of surgery**		
*Hepatobiliary*	11 (26.8)	3 (8.1)
*Urology*	8 (19.5)	9 (24.3)
*Breast and endocrine*	7 (17.1)	8 (21.6)
*Orthopedic*	5 (12.2)	5 (13.5)
*Vascular*	3 (7.3)	7 (19.0)
*Colorectal*	5 (12.2)	2 (5.4)
*Gastroenterology*	1 (2.4)	3 (8.1)
*Plastics*	1 (2.4)	0 (0)
**ASA physical status**		
*I*	3 (7.3)	5 (13.5)
*II*	19 (46.3)	17 (46.0)
*III*	19 (46.3)	15 (40.5)
**Preoperative waiting time (mins)**	128.5 (79.5-181.5)	138 (99-211)
**Duration of surgery (mins)**	121(90-202)	106 (67-178)
**PACU length of stay (mins)**	70 (54-110)	55 (36-80)

*Note*: Median (IQR) or no. (%). *Abbreviations*: ASA American Society of Anesthesiologists

### Feasibility

Table 2 reports feasibility outcomes including recruitment, retention, treatment adherence and missing or incomplete data. Inability to contact potential participants for recruitment was the major cause of non-enrolment after screening for eligibility. Retention is reported for the entire study cohort (*n* = 102). There were no adverse events related to the use of the ZHF device (e.g., skin reaction to the sensor).

**Table 2:** Feasibility outcomes

**Feasibility outcomes**	***n* (%)**
**Recruitment**	
*Enrolled versus screened participants*	102/170 (60)
*Total non-enrolled participants*	68/170 (40)
**Reasons for non-enrolment**	
*Unable to contact potential participants*	34/68 (50)
*Declined*	13/68 (19.1)
*Surgery cancelled*	9/68 (13.2)
*Unable to consent preoperatively*	6/68 (8.8)
*In-patient*	3/69 (4.4)
*No general anesthetic planned*	1/69 (1.5)
*Unable to consent due to mental capacity*	1/69 (1.5)
*Planned postoperative ICU admission*	1/69 (1.5)
**Retention**	
*Drop-outs due to cancellation after enrolment*	98/102 (96)
**Treatment adherence (ZHF group)**	
*Preoperative phase (Surgical Care Unit)*	37/40 (92.5)
*On arrival to anesthetic room*	27/37 (73.0)
*On induction*	16/37 (43.2)
*During surgery*	24/37 (64.9)
*On PACU arrival*	25/37 (67.6)
**ZHF data missing/incomplete**	
*Preoperative phase (Surgical Care Unit)*	3/37 (8.1)
*On arrival to anesthetic room*	8/37 (21.6)
*On induction*	19 (51.4)
*During surgery*	11 (29.7)
*On PACU arrival*	10 (27.0)
*During PACU*	10 (27.0)

*Abbreviations: ZHF* Zero-heat-flux, *PACU* Post Anesthetic Care Unit

Incomplete or missing temperature data in the ZHF group is detailed in Table 2: cases are reported where the ZHF device was applied and in-use but data did not transfer in full into the EMR. In two cases, the protocol was not adhered to across all timepoints (from device attachment in the SCU through the intraoperative and PACU phases). In two cases, the device was not applied in SCU but was applied in the anesthetic room, as the patient was called to surgery early.

### Staff evaluation

Of the 23 health professionals that proceeded from the email containing the link to the participant Information and survey, 16 (69.6%) completed the staff survey. Of these, seven (44%) were registered nurses (five anesthetic nurses, two PACU nurses) and nine (56%) were anesthesiologists (four registrars and five consultant anesthesiologists). Half had used the ZHF device: assessment of acceptability, intervention appropriateness, feasibility and practicality are reported in Table 3. Median values indicated that the ZHF device was considered acceptable, appropriate, feasible and practical. Survey respondents who had not yet used the ZHF device were asked if they would consider using the device: six of eight respondents stated that they would consider using the ZHF in their future practice. Two anesthesiologists responded that they would not. Of those who had used the device, 75% scored neutral or higher on all survey items.

**Table 3:** Health professional evaluation of the ZHF device (*n* = 8)

**Survey items**	**Median (IQR)**
Acceptability of Intervention (AIM)	3.9 (2.4-4.3)
Intervention Appropriateness Measure (IAM)	3.8 (2.5-4.5)
Feasibility of Intervention Measure (FIM)	3.9 (2.5-4.3)
Practicality	4 (3-4)

### Process of care evaluation

Proportions of temperature monitoring at any timepoint for both groups are reported in Table 4. Additionally, proportions of temperature monitoring meeting guideline recommendations were assessed. Guidelines are that temperature should be measured in the hour prior to surgery [3, 4]. We reported temperature on arrival to the anesthetic room as this reflects the process of care in the study hospital: this increased from zero in the usual care to three patients in the ZHF group. Intraoperatively, as per guidelines [3, 4], monitoring is reported as every 30 minutes (for surgery duration of > 30 minutes) and every 15 minutes (for patients receiving forced air warming). In PACU, temperature is reported as per guidelines on admission, every 15 minutes and at discharge [3, 4]: the largest difference was found between groups for patients receiving monitoring every 15 mins in PACU. In the usual care group, zero patients received monitoring at these timepoints, versus 17 (46%) of patients in the ZHF group.

**Table 4:** Process of care outcomes

**Process of care outcomes**	**Usual care** **(*n* = 41)**	**ZHF group** **(*n* = 37)**
**Temperature monitoring (any timepoint)**		
*Intraoperative*	21 (51.2)	17 (46.0)
*PACU*	41 (100)	35 (94.6)
**Temperature monitoring meeting guidelines**		
***Preoperative***		
*Arrival to anesthetic room*	0 (0)	3 (8.1)
*Induction*	0 (0)	5 (13.5)
*** Intraoperative***		
*Every 30 mins (without forced air warming)*	8/13 (61.5)	5/11 (45.5)
*Every 15 mins (with forced air warming)*	3/28 (11)	5/26 (19.2)
*** PACU***		
*On admission*	38 (92.7)	30 (81.1)
*Every 15 minutes*	0 (0)	17 (46)
*On discharge*	2 (4.9)	10 (27.0)
**Warming interventions**		
***Preoperative***	1 (2)	0 (0)
*Forced air warming*	1	0
***Intraoperative***	30 (73.1)	29 (78.4)
*Warmed cotton blankets*	2	10
*Forced air warming*	28	26
*Warmed intravenous fluids*	0	3
***PACU***	2 (5)	0 (0)
*Warmed cotton blankets*	2	0
**Any temperature monitoring intraoperatively with forced air warming**	15/28 (53.6)	15/26 (57.7)
**Hypothermia on PACU admission**		
*Normothermia (≥ 36°C)*	24 (63.2)	15 (50)
*Mild (35.0-35.9°C)*	13 (34.2)	13 (43.3)
*Moderate (34.0-34.9°C)*	1 (2.6)	1 (3.3)
*Severe (<33.9°C)*	0 (0)	1 (3.3)

*Abbreviations: PACU* Post Anesthetic Care Unit

**Fig. 2:**
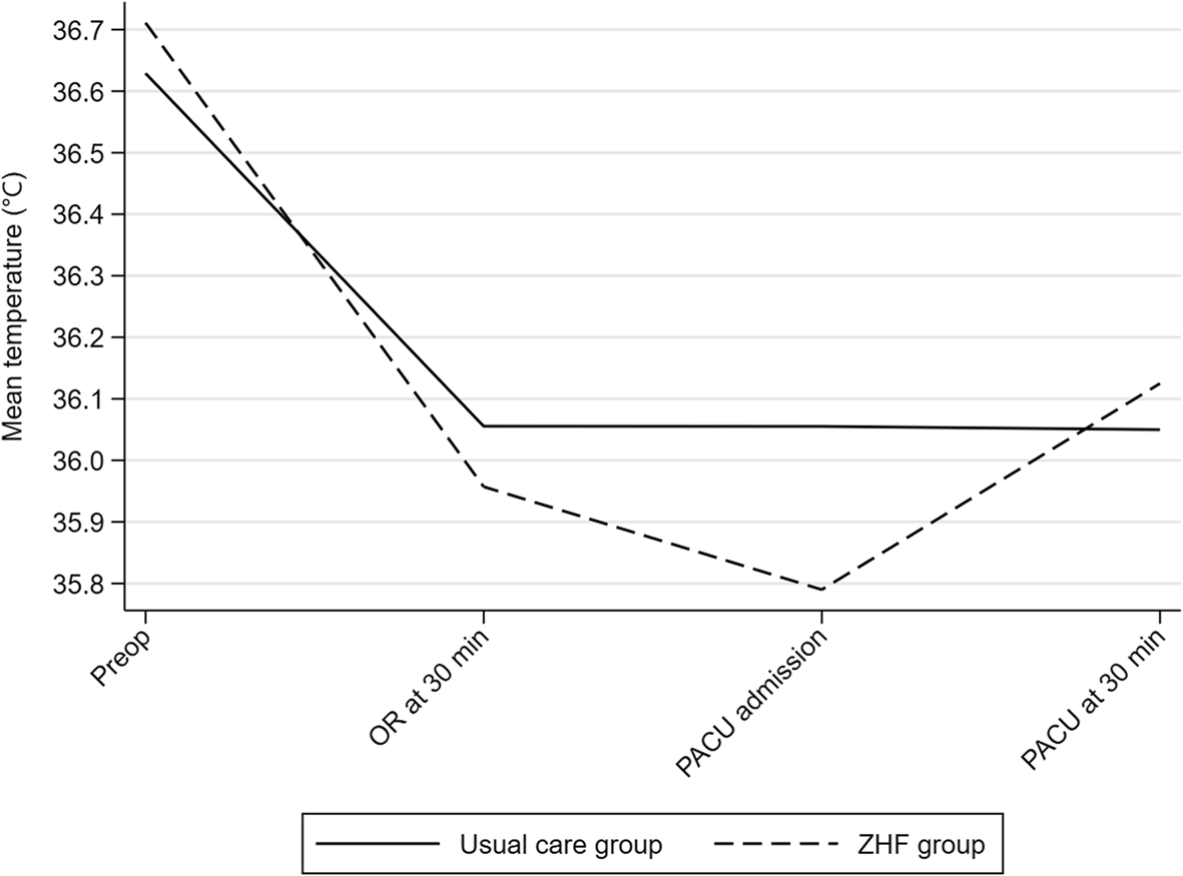
Mean temperatures by group from preoperative phase to 30 minutes in PACU. *Abbreviations*: OR: operating room; PACU: Post Anesthetic Care Unit: ZHF: zero-heat-flux

On PACU admission, mean body temperature adjusted for baseline preoperative temperature and BMI was 36.03°C in the before group and 35.86°C in the ZHF monitoring group (adjusted mean difference = -0.17; 95% CI -0.49, 0.15). Proportions of mild, moderate, and severe hypothermia by group on PACU admission are presented in Table 4 (Fisher’s exact test, *p* = 0.59). Figure 2 presents mean temperature decline from the preoperative phase to 30 minutes in PACU.

## Discussion

In this single-center feasibility study we found the introduction of continuous ZHF monitoring throughout perioperative care improved temperature monitoring practices during post-anesthetic care but had limited uptake intraoperatively. Feasibility of recruitment in preparation for a larger trial was established. Yet, strategies to improve protocol adherence and to reduce missing data across the multiple time points included in the study protocol are needed.

We were interested in whether the provision of a device that was already in place on arrival to the OR would increase intraoperative monitoring as per guidelines [3, 4, 24]. Temperature monitoring adherence only slightly increased at *on admission* and *on induction* timepoints. The greatest improvements in adherence to monitoring guidelines were found during PACU care, where no patients received regular 15-minute monitoring in the usual care group versus 17/37 (46%) in the ZHF group. Yet improvements in pre- and intra-operative temperature monitoring are hypothesized to have the greatest impact on clinical outcomes by enabling pre-emptive action to prevent heat loss [11].

In line with earlier research indicating that the continuous ZHF device itself is acceptable to patients [17, 24], only a small number of patients approached for consent declined to participate. The major issue affecting feasibility of a larger study is treatment adherence: not only staff adherence to the protocol, but the reliability of electronic data transfer from the device to the EMR. In a future trial, additional safeguards such as prospective, real-time data collection are needed to mitigate the risk of missing data due to transfer issues, as experienced in this and earlier studies [14, 17]. An implementation design that accounts for the complexity of practice change across perioperative systems and interprofessional teams is needed [25]. However, these strategies will increase the costs of a large-scale trial.

Basic improvements to the design of the ZHF may enhance clinical uptake. The device requires mains power to function, impacting on ease of use during transfer. Time for the device to achieve equilibrium on being plugged in on arrival to a new area affects consistency of monitoring, as acknowledged in other studies [26]. In our study, the time for equilibrium may have caused missing data at some timepoints, including *on induction* and *on PACU admission*. At PACU admission, temperature measurement typically occurs immediately during patient arrival during handover. For the ZHF group the time required to reach equilibrium may account for missing measurements during care transitions such as induction and PACU admission.

Improved integration of the ZHF device into existing monitoring devices and processes could maximize the usefulness of continuous temperature monitoring. The device retains the last two hours of data, yet this can only be displayed graphically (without raw values). Plugging the device into the anesthetic monitor can allow for retrieval of raw values. Yet, despite attachment to the monitor, we experienced data transfer issues where the data could not be retrieved from the EMR. Enhancing the device to improve data retention and addressing connectivity and data transfer issues would be of benefit to both routine practice and for research purposes.

### Limitations

Staff evaluation of ZHF monitoring acceptability, feasibility, appropriateness and practicality indicated that the device was generally favorable. However, our conclusions here are cautious due to a limited response to the survey assessing these attributes. While the survey was distributed via email, planned paper-based surveys were prevented as data collection occurred during several periods of COVID-19 lockdown and restrictions. This limited the response rate.

Within this setting, the surgical population was not homogenous: we enrolled patients from a variety of surgical specialties. A larger trial might benefit by stratifying the sample by surgical specialty, taking into account factors that may alter decision-making regarding temperature monitoring for particular subgroups of patients or procedures. The use of the device may be limited for those patients’ undergoing neurosurgery or facial surgery. Although designed for use on the forehead, the device has been further evaluated for use in neck and chest regions, suggesting alternative sites with acceptable reliability in these populations [27, 28].

## Conclusion

Improved integration of the ZHF device into existing electronic systems is needed prior to a large-scale evaluation of whether continuous monitoring reduces the number of patients exposed to perioperative hypothermia. To our knowledge, the larger study piloted here would be the first to assess the impact of continuous temperature monitoring on perioperative hypothermia prevention practices and patient outcomes. 

## Data Availability

The datasets used and/or analysed during the current study are available from the corresponding author on reasonable request.

## Abbreviations

AIM: Acceptability of Intervention Measure
ASA: American Society of Anesthesiologists
BMI: Body Mass Index
EMR: Electronic Medical Record
FIM: Feasibility of Intervention Measure
IAM: Intervention Appropriateness Measure
ICU: Intensive Care Unit
OR: Operating Room
PACU: Post Anesthetic Care Unit
SCU: Surgical Care Unit
ZHF: zero-heat-flux
